# Development of a Bioluminescent Nitroreductase Probe for Preclinical Imaging

**DOI:** 10.1371/journal.pone.0131037

**Published:** 2015-06-25

**Authors:** Anzhelika G. Vorobyeva, Michael Stanton, Aurélien Godinat, Kjetil B. Lund, Grigory G. Karateev, Kevin P. Francis, Elizabeth Allen, Juri G. Gelovani, Emmet McCormack, Mark Tangney, Elena A. Dubikovskaya

**Affiliations:** 1 School of Basic Sciences, Institute of Chemical Sciences and Engineering, Swiss Federal Institute of Technology of Lausanne, Lausanne, Switzerland; 2 Cork Cancer Research Centre, University College Cork, Cork, Ireland; 3 Department of Clinical Science, University of Bergen, Bergen, Norway; 4 Department of Internal Medicine, Hematology Section, Haukeland University Hospital, Bergen, Norway; 5 PerkinElmer, Hopkinton, Massachusetts, United States of America; 6 School of Life Sciences, Swiss Institute for Experimental Cancer Research (ISREC), Swiss Federal Institute of Technology of Lausanne, Lausanne, Switzerland; 7 Department of Biomedical Engineering, College of Engineering and School of Medicine, Wayne State University, Detroit, Michigan, United States of America; Ecole Polytechnique Federale de Lausanne, SWITZERLAND

## Abstract

Bacterial nitroreductases (NTRs) have been widely utilized in the development of novel antibiotics, degradation of pollutants, and gene-directed enzyme prodrug therapy (GDEPT) of cancer that reached clinical trials. In case of GDEPT, since NTR is not naturally present in mammalian cells, the prodrug is activated selectively in NTR-transformed cancer cells, allowing high efficiency treatment of tumors. Currently, no bioluminescent probes exist for sensitive, non-invasive imaging of NTR expression. We therefore developed a "NTR caged luciferin" (NCL) probe that is selectively reduced by NTR, producing light proportional to the NTR activity. Here we report successful application of this probe for imaging of NTR *in vitro*, in bacteria and cancer cells, as well as *in vivo* in mouse models of bacterial infection and NTR-expressing tumor xenografts. This novel tool should significantly accelerate the development of cancer therapy approaches based on GDEPT and other fields where NTR expression is important.

## Introduction

The nitroreductase (NTR) family of enzymes are widespread amongst bacteria and are known to metabolize nitrosubstituted compounds and quinones using NADH or NADPH as reducing agents [1–4]. They are important for the development of novel antibiotics being the main target for the treatment of infections caused by bacteria, e.g. *Mycobacterium tuberculosis* [5], *Helicobacter pylori* [6] and by parasites, e.g. Trypanosoma [7], Giardia and Entamoeba [8]. Their enzymatic activity in gut microbiota is linked to carcinogen production and etiology of colorectal cancer [9,10]. In addition, they are used in biotechnology for degradation of environmental contaminants [1]. Due to their absence in mammalian cells they are also utilized as activating enzymes in gene-directed enzyme prodrug therapy (GDEPT) approaches for cancer chemotherapy [11] where the NTR gene is used to selectively transform cancer cells, providing unique targeted therapy of tumors over normal tissues [12]. Nitroaromatic prodrug CB1954 in the complex with bacterial NTR is promising for GDEPT and has reached clinical trials for prostate cancer [13].

Following the recent first approval in Europe of a gene therapy medicine, the potential for clinical application of GDEPT is increasing [14]. However, both preclinical and clinical development of NTR-based GDEPT systems has been severely hampered by the lack of imaging tools that allow sensitive *in vivo* evaluation of transgene expression in living subjects. Quantification of the level of transgene expression is extremely important because it is directly linked to the effectiveness of the therapy.

Bioluminescence (BL) is currently the most sensitive optical in vivo imaging modality available, and has been applied to visualize multiple biological processes in small animals [15,16]. It obviates most of the limitations of *in vivo* fluorescent imaging [17], such as high tissue-derived autofluorescence, photobleaching, limited tissue penetration and lack of quantification. Several activatable fluorescent probes for NTR imaging in vitro have been previously described [18–22]. However, the only reported probe for imaging of NTR *in vivo* relies on fluorescence (CytoCy5S) [23–25] and therefore possesses the limitations mentioned above.

Similar to bioluminescent imaging, *in vivo* chemiluminescent imaging offers the advantages of high sensitivity due to low background and high signal-to-noise ratios. Prior studies have elegantly demonstrated the application of chemiluminescence for imaging of myeloperoxidase activity [26] and beta-galactosidase activity [27] *in vivo*. However, although chemiluminescence has the additional advantage of not requiring luciferase transfected cells for the generation of light, expression of this enzyme allows more elaborate disease models to be developed due to the researcher’s ability to define its spatial localization and regulation. Moreover, most chemiluminescent agents suffer from low quantum yield, short maximal photon wavelength emission and high instability. For example, the quantum yield of aqueous luminol chemiluminescence is 1.23 ± 0.20% [28] with a maximal emission of 424 nm [29], while the reported quantum yield of firefly BL is 41.0 ± 7.4% [30], that is about 40 times higher, with D-hydroxyluciferin and D-aminoluciferin having wavelength at 560 nm and 603 nm respectively [31]. Recently, Zhang *et al*. [32] showed the advantage of using both near-infrared fluorescent and chemiluminescent imaging in combination, while addressing the wavelength issue associated with luminol chemiluminescence *in vivo* by shifting it into the near-infrared region utilizing quantum dots.

BL is based on the interaction of a small molecule D-luciferin with firefly luciferase that results in the generation of photons of light. The system can be "tuned" through "caging" of the luciferin scaffold to image and quantify activities of biological molecules. Target-mediated selective removal of the caging moiety leads to production of free D-luciferin and subsequent generation of photons by luciferase, that can be quantified [33]. While this strategy was previously used by us and others to study biological processes (delivery and biodistribution of cell-penetrating peptide conjugates [34], cell surface glycosylation [35], hydrogen peroxide fluxes [36], fatty acids uptake [37]) and image enzyme activity (beta-galactosidase [38], caspases [39–41], furin [42] and beta-lactamase [43], no bioluminescent probes have been previously reported for imaging of NTR. Here, we describe the development of novel NTR-specific bioluminescent probe, termed Nitroreductase Caged Luciferin (NCL). Our results demonstrate that this probe can be used for non-invasive real-time imaging of NTR activity *in vitro*, in live bacteria and mammalian cells, as well as in preclinical models of cancer and bacterial infection.

## Materials and Methods

### Chemical materials and synthesis

The synthetic procedures and characterization are detailed in the S1 File.

### Kinetics of NCL reaction with NTR by fluorescence

Fluorescence was measured using a Tecan Infinite M1000 (Tecan Austria GmbH) plate reader. Kinetic measurements of NCL (5–50 μM) uncaging by NTR (0.25 μg mL^-1^) were performed in the presence of NADH (500 μM) at 37°C in PBS buffer (pH 7.4). The kinetics rate of luciferin release from NCL was measured by fluorescence at 330 nm excitation and 530 nm emission wavelengths. The fluorescence calibration curve for luciferin was used to calculate the rate (S2 Fig). Kinetic parameters K_m_ and V_max_ were determined from Michaelis-Menten model and Lineweaver-Burk plot was used to display the data. The k_cat_ value was calculated by dividing the V_max_ value, obtained from the data acquired for the determination of the corresponding K_m_ values for the probe, by the concentration of the nitroreductase in the assay.

### Inhibition assay of NTR by dicoumarol in enzyme assay

NCL (20 μM) uncaging by NTR (0.5 μg mL^-1^) in the presence of NADH (100 μM) was inhibited by various concentrations of dicoumarol (0 to 200 μM). Luciferin release from NCL was measured by fluorescence at 330 nm excitation and 530 nm emission wavelengths over time. Inhibitory activity of dicoumarol is expressed as a percentage compared to uninhibited control (S3 Fig).

### Bioluminescent imaging of NTR with NCL in enzyme assay

*In vitro* imaging studies were performed in clear bottom black 96 well plates from Becton Dickinson and Company. An IVIS Spectrum (PerkinElmer) was used to measure the amount of bioluminescent imaging (BLI) signal production. The data are presented as pseudocolor images indicating light intensity (red being the most intense and blue the least intense), which are superimposed over the grayscale reference photographs. Bioluminescence was quantified using region of interest (ROI) analysis of individual wells and the average signal expressed as the total number of photons emitted per second per cm^2^ per steradian (p/sec/cm^2^/sr) from each of the three wells was calculated by using the Living Image software. Total luminescence was calculated by integrating the area under corresponding kinetic curves. Luciferase buffer was prepared as following: 2 mM ATP, 5 mM MgSO_4_ in PBS (pH 7.4). Stock solutions of luciferase in luciferase buffer, NADH, NCL, luciferin and NTR in PBS (pH 7.4), were freshly prepared and aliquoted in a 96-well plate to give the following final concentrations in the total volume of 100 μL/well: luciferase (60 μg mL^-1^), NADH (100 μM), NTR (10 μg mL^-1^), luciferin or NCL (0.25–5 μM); luciferin and NCL were added at the last step with a multichannel pipette from the additional 96-well plate. Bioluminescence signal from the plate was acquired immediately every 1 min with 0.5 s integration time for 30 min.

### Bacterial strains, plasmids and culture conditions

*E*. *coli* K-12 MG1655 (- luc gene) and *E*. *coli* K-12 AB1157, containing the luciferase expressing pUC57 Click beetle red (CBR) plasmid (+ luc gene) was a kind gift from Daniel Ansaldi (Perkin Elmer). The NTR triple mutant, *E*. *coli* K-12 AB502NemA, was a kind gift from Dr. Antonio Valle (University of Cádiz, Cádiz, Spain) and was transformed with the pUC57 CBR plasmid for production of luciferase. *E*. *coli* MG1655 lux, expressing lux luciferase, was generated as previously described [44]. All strains were grown aerobically at 37°C in Luria Bertani (LB) medium supplemented with 100 μg mL^-1^ ampicillin (Amp).

### Bioluminescent imaging of NTR activity by NCL in *E*. *coli*

An IVIS-100 (PerkinElmer) was used to measure the amount of BLI signal production. Stock solutions of luciferin and NCL in PBS (pH 7.4) were freshly prepared and aliquoted in a 96-well plate to give the final concentrations (1–250 μM) in the total volume of 200 μL/well. The volume of bacterial suspension was 150 μL/well. Bioluminescence signal from the plate was acquired immediately every 2 min with 10 s integration time for 1 h.

### Cell lines and cell culture

Cell line MDA-MB-231-NTR-Fluc-EGFP (NTR+luc+) used for *in vitro* and *in vivo* experiments in this study was kindly provided by Dr. Ramasamy Paulmurugan (Stanford University School of Medicine, Stanford, USA) [25]. The cells were generated as described below with a reference to Sekar et al. [25]. The cloning vectors, expressing bacterial nitroreductase gene (NTR2) and Fluc-EGFP fusion constructs, were from the plasmid bank (Cellular Pathway Imaging Laboratory, Stanford). To make MDA-MB-231 stable cell line, modified pcDNA3.1 (PURO) vector expressing NTR was transfected using Lipofectamine 2000 Transfection Reagent (Invitrogen). 24 hours later medium was changed and the cells were treated with 100 ng mL^-1^ of puromycin. The process was continued until no further cell death was observed. The cells were plated in low dilution (1 cell/100 μL) in a 96 well plate. Single colonies of cells expressing NTR were expanded for further transduction with lentivirus expressing Fluc-EGFP fusion protein. To control the level of Fluc-EGFP at near equal expression, cells were sorted by FACS in a similar window after transduction. MDA-MB-231 stable cells were maintained in puromycin stress throughout the study. Single colonies of stable cells were evaluated for the functionality of NTR enzyme by incubating with a CytoCy5S (red-shifted NTR substrate; GE Healthcare) for the detection of fluorescent signal (λ_ex/em_ = 628 nm/ 638 nm).

Cell line MDA-MB-231-luc-D3H2LN Bioware (NTR-luc+), used as NTR negative control for *in vitro* and *in vivo* experiments in this study, was purchased from PerkinElmer, and maintained in Dulbecco’s Modified Eagle’s Medium supplemented with 10% (v/v) heat-inactivated FBS, 1% (v/v) penicillin/streptomycin (all reagents purchased from Life Technologies).

### Bioluminescent imaging of NTR by NCL in stable cell lines

MDA-MB-231-NTR-Fluc-EGFP cells and control MDA-MB-231-luc cells were plated at a density 3 × 10^4^ cells/well in two black 96-well plates with clear bottom, after 48 h the growth medium was removed, and 100 μL of NCL probe or luciferin solutions (1–100 μM) in cell culture medium was added to the wells. The plates were immediately placed in IVIS Spectrum and were imaged every 1 min for 1 h. Observed BLI signal was quantified using ROI analysis with Living Image software.

### Ethics statement

All animal procedures on imaging of bacterial NTR in a mouse model of thigh infection were performed in accordance with the national ethical guidelines prescribed by the Health Products Regulatory Authority (HPRA). Protocols were approved by the animal ethics committee of University College Cork (AERR #2010/003 and #2012/015). Experiments on imaging of NTR in a mouse model of subcutaneous cancer were carried out in strict accordance to the Swiss regulation on animal experimentation and the protocol (#2363) was approved by the authority of the Canton Vaud, Switzerland (EXPANIM (Expérience sur animaux)–SCAV, Département de la sécurité et de l’environnement, Service de la consommation et des affaires vétérinaires). All efforts were made to minimize suffering.

### Bacterial administration and imaging of bacterial nitroreductase in mice

Bacteria were grown at 37°C in a shaking incubator until reaching OD_600_ of 0.6 in LB medium, containing 100 μg mL^-1^ Amp. Cultures were harvested by centrifugation (4000 × g for 10 min) and washed three times in PBS. After washing, bacteria were resuspended in one tenth volume of PBS. Mice were kept at a constant room temperature (22°C) with a natural day/night light cycle in a conventional animal colony. Standard laboratory food and water were provided ad libitum. Mice were afforded an adaptation period of at least 7 days before the beginning of experiments. Female BALB/c mice (Harlan, Oxfordshire, UK) in good condition, without infections, weighing 18–22 g and 6–8 weeks old, were kept as previously described [45] and were included in experiments. BALB/c mice were anaesthetized and the fur on the rear legs was removed. Mice were injected directly into the right quadriceps muscle at a depth of approximately 5 mm with 50 μL of bacteria suspended in PBS. The concentration of bacterial suspensions used for injection ranged from 10^6^ to 10^9^ bacteria/mL. Mice also received an intramuscular injection of 50 μL sterile PBS in the left rear quadriceps (control) and lux MG1655 *E*. *coli* (positive control). 1 h post bacterial injection, mice received an IP injection of 200 μL of 10 mM NCL probe (0.8 mg) or 200 μL of 10 mM D-luciferin potassium salt (0.63 mg) in PBS. For *in vivo* experiments involving varying concentrations of the probe, the concentration of NCL probe injected was 200 μL of 1, 10 or 20 mM solutions in PBS (0.08, 0.8 or 1.6 mg of NCL probe). Mice were imaged for bioluminescence at regular intervals beginning immediately after probe injection using IVIS 100. Mice that were infected with bacteria for experimental purposes were monitored for signs of illness for the duration of the experiment. No adverse symptoms were reported. Following bioluminescence imaging, or at experiment end, animals were euthanized by cervical dislocation.

### Mice and tumor induction

Swiss nu/nu mice were obtained from Charles River Labs. Mice were maintained at the EPFL UDP animal facility under pathogen free conditions and group housed in individually ventilated cages at 22°C with 12/12 light cycle. Before experiments the mice were afforded an adaptation period of at least 7 days. Female mice in good condition, weighing 18–25 g and 6 weeks of age were randomly divided in two experimental groups (n = 5 per group). For tumor induction, 1 × 10^6^ cells in 100 μL of FBS-free medium/Matrigel (BD Biosciences) (50:50) was injected subcutaneously into the flank of the mice. One group was injected with MDA-MB-231-NTR-Fluc-EGFP cells and the other group- with MDA-MB-231-luc cell (control). The viability of cells used for inoculation was more than 95% as determined by Trypan Blue Dye Exclusion (Gibco). Following tumor establishment, the health parameters that may lead to the endpoints were carefully monitored in xenograft mice three times a week: cachexia (acute weight loss), lack of activity and loss of appetite. Weight was measured and general behavior as well as body conditions was assessed. Tumor size was carefully monitored to ensure that it doesn’t exceed maximal allowed size of 1 cm^3^. Tumor volume was measured by caliper and calculated according to the formula 1/2(length × width^2^). When tumors reached approximately 0.1 cm^3^ in volume, the mice were imaged with luciferin (1.5 mg/mouse in 50 μL of PBS IP) for 1 h once a week to estimate the light emission and the optimal imaging window. Bioluminescence was acquired using IVIS Spectrum, every 1 min for 1 h with the auto-exposure mode. Following bioluminescence imaging at experiment end animals were euthanized by CO_2_ inhalation or cervical dislocation.

### Imaging of nitroreductase in a mouse model of subcutaneous cancer

Potassium salt of D-luciferin (Intrace Medical) was dissolved in PBS (pH 7.2), solution was filter sterilized through 0.22 μm filter, aliquoted and kept at –20°C. NCL probe was dissolved in PEG400 (Sigma-Aldrich) and diluted with sterile PBS (pH 7.2) 1:5 (20% (v/v) of PEG400 in the total volume of 200 μL), fresh solution was prepared before every imaging. The dose of NCL probe (1.9 mg/mouse) was equivalent (4.7 μmol) to the dose of luciferin (1.5 mg/mouse). Mice were anesthetized prior to injection and during imaging via inhalation of isoflurane (Piramal Critical Care, Inc). When the total photon flux over 1 h from the mice imaged with luciferin reached 1 × 10^8^, the mice entered the experiment. On day 1 of the experiment all mice were injected IP with luciferin (1.5 mg/mouse in 50 μL of PBS) and bioluminescence was acquired immediately every 1 min for 1 h with the auto-exposure mode. On day 2 of the experiment (24 h after the luciferin imaging) all mice were injected IP with NCL probe (1.9 mg/mouse in 200 μL of PBS containing 20% (v/v) PEG400) and bioluminescence was acquired immediately every 1 min for 1 h with the auto-exposure mode.

### Data analysis from a cancer model experiment

Bioluminescence was quantified using ROI analysis of the tumor area individually for each mouse. Total luminescence was calculated by integrating the area under corresponding kinetic curves. Percent of the NCL probe uncaging was calculated individually for each mouse using the formula:

$$\%\;of\;uncaging=\;\frac{Total\;photon\;flux\;over\;1\;h\;from\;NCL\;\times \;100\%}{Total\;photon\;flux\;over\;1\;h\;from\;luciferin}$$

### Statistical analysis

Two-tailed Student’s t-test was used to determine statistical significance (GraphPad Prism 6.03, GraphPad Software).

## Results and Discussion

### Probe design

The reduction of nitroaromatic compounds can occur through one- or two-electron mechanism [1]. Two types of bacterial NTRs have been described and they are classified according to the oxygen dependence. The NTRs used in our study (NfsA, NfsB) are type I oxygen-insensitive NTRs, they catalyze the reduction of the nitro group by addition of a pair of electrons, and their activity does not depend on the level of oxygen. However, the oxygen-sensitive NTRs (type II) catalyze the reduction of the nitro group by the addition of one electron, forming the nitro anion radical, which is oxidized back to the nitro group by oxygen. Bacteria contain both types of nitroreductases with type I being the most characterized among other NTRs. The independence of reduction from the level of oxygen in *E*. *coli* had been previously demonstrated for an NTR-sensitive coumarin probe (7-nitrocoumarin-3-carboxylic acid) suggesting the prevalent involvement of type I NfsA and NfsB possibly along with other uncharacterized NTRs [46].

We have previously demonstrated the suitability of exploiting NTR activity as being sufficiently selective to distinguish bacterial cells from host background [47]. Several NTR-related enzymes have been identified in mammalian cells and they functionally relate to type I NTRs (NAD(P)H-quinone oxidoreductase (DT-diaphorase EC 1.6.99.2) and xanthine dehydrogenase EC 1.17.1.4). They can potentially contribute to reduction of nitroaromatics, although they are not phylogenetically related and do not exhibit the typical domain characteristic of NTR family. These properties were investigated in the study on FMISO imaging reagent, a derivative of nitroimidazole used as a hypoxia PET tracer [48]. It was reported that under hypoxic conditions xanthine dehydrogenase is converted to xanthine oxidase that reduces FMISO and other nitroimidazole-containing compounds. Similarly, eukaryotic NTRs that are functionally related to type II (aldehyde oxidase EC 1.2.3.1, cytochrome c oxidase EC 1.9.3.1, and NADPH cytochrome P450 reductase EC 1.6.2.4) can potentially reduce nitroaromatics anaerobically and are generally used as targets for hypoxia-activated prodrugs and imaging agents [49].

Therefore, several important factors need to be taken into account when designing the compounds activated selectively by bacterial or mammalian enzymes. For bacterial NTRs the activation is largely dependent on the redox potential of the nitroaromatics. For example, nitrofurans display relatively high redox potentials (reported from -250 to -270 mV) and are reductively activated by NAD(P)H nitroreductases of enteric bacteria. At the same time metronidazole (nitroimidazole) is only activated by anaerobic enzymes showing low redox potentials (-480 mV) in some bacteria and protozoa, making it well tolerated in humans when used as an antibiotic [50].

Substrate specificity of the designed compounds is also important, for example CB 1954 prodrug is efficiently reduced by bacterial NTRs and a human DT-diaphorase (NQO2) while being a poor substrate for a human paralogue NQO1 enzyme [51]. These and other important aspects of selectivity of bioreductive prodrugs are discussed in more details in a recent review by Wilson and Hay [52].

**Fig 1:**
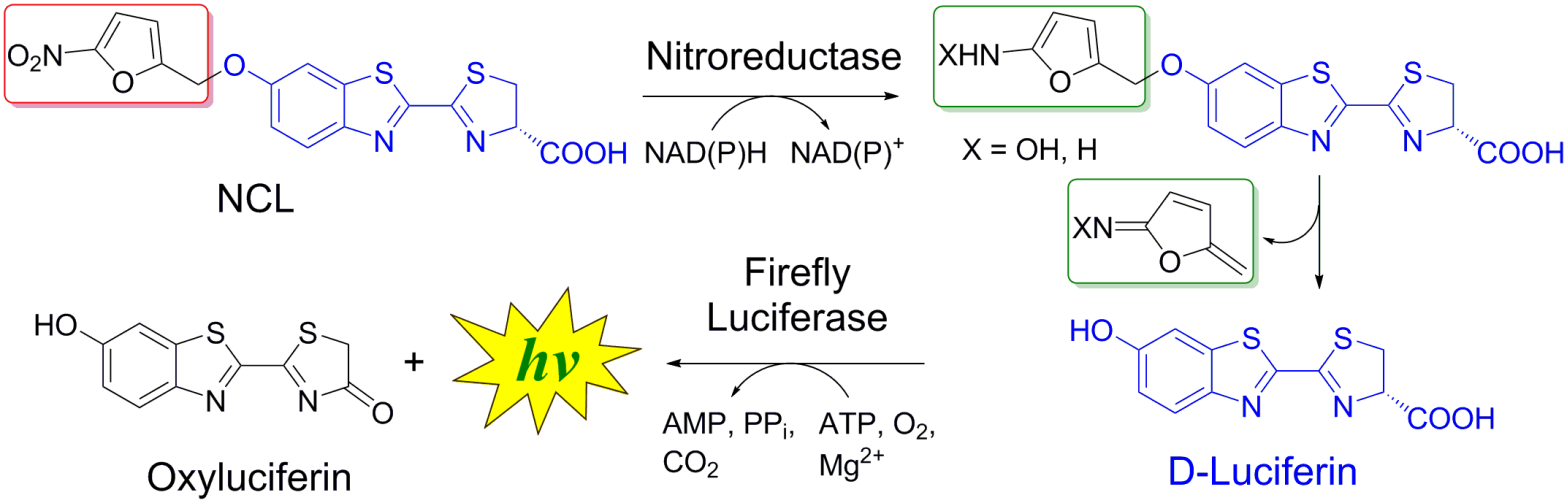
General strategy for imaging of NTR activity with Nitroreductase Caged Luciferin (NCL) probe.

The overall probe design is based on caging of D-luciferin with nitrofuryl moiety resulting in "Nitroreductase Caged Luciferin" (NCL) probe (Fig 1). 5-Nitrofuryl was selected as a cage as its derivatives (nitrofurazone, nitrofurantoin) were shown to be efficiently activated by NTR in bacteria [50]. Upon the reduction of the nitro group by NTR the resulting electron-donating amino group promotes the cleavage of the C-O bond (uncaging), leading to the subsequent release of luciferin which is oxidized by luciferase and a photon of light is emitted. Therefore, release of free luciferin followed by light production is only possible in the presence of NTR.

### Bioreductive activation of NCL in cell-free assays

We first investigated the specificity of probe uncaging by incubating NCL with a recombinant NTR enzyme from *E*. *coli* (NfsA) in the presence of NADH as a cofactor. The release of luciferin was followed by HPLC-MS analysis as a function of time (S1 Fig). The resulting data demonstrated rapid conversion of NCL into free luciferin under these conditions, the calculated values of rate constant and NCL half-life, were 5.8 × 10^-3^ s^-1^ and 119.5 s respectively (S1 Fig).

**Fig 2:**
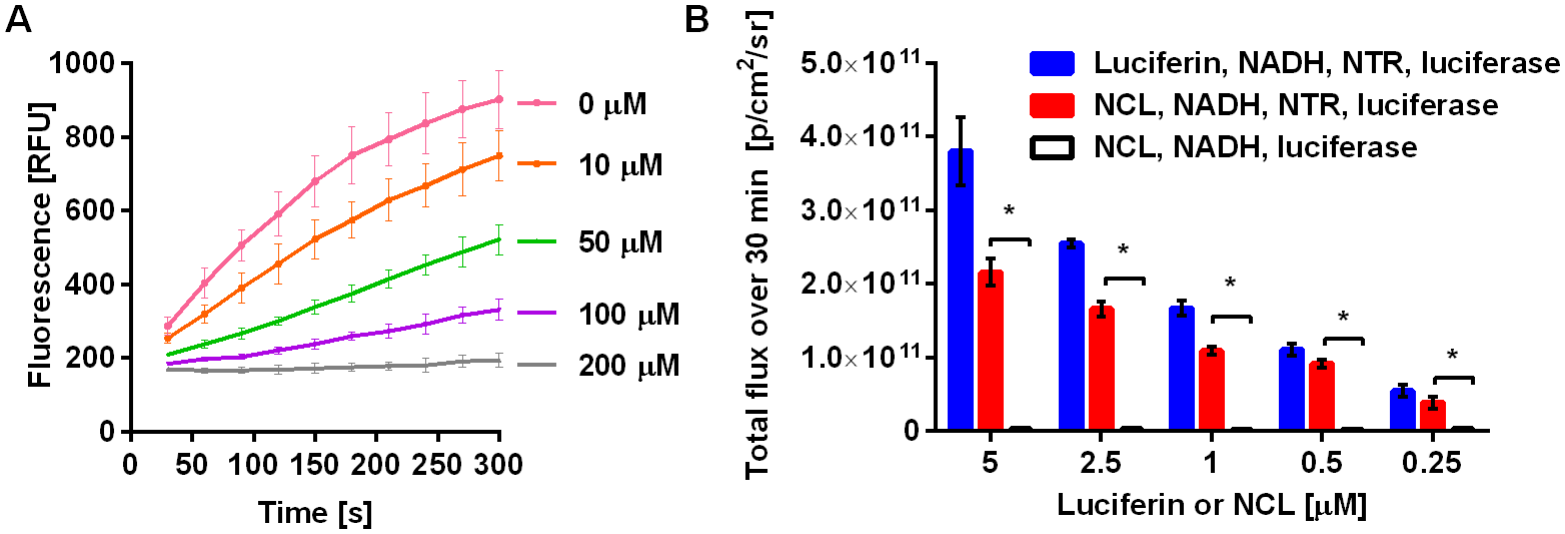
Evaluation of NTR-specific uncaging of NCL probe. (A) NCL (20 μM) uncaging by NTR (0.5 μg mL^-1^) in the presence of NADH (100 μM) was inhibited by dicoumarol (0 to 200 μM). (B) Total luminescence over 30 min from luciferin or NCL (0.25–5 μM) with NADH, luciferase, and NTR, compared with the control (no NTR), *P < 0.001.

Next, we determined the Michaelis-Menten kinetic parameters for the NTR-specific cleavage of NCL probe (S2 Fig) using fluorescence to monitor release of luciferin (λ_ex/em_ = 330/530 nm) as the caged probe is not fluorescent. Both V_max_ and K_m_ values were found to be comparable to those previously reported for a NTR fluorescent substrate [20] and were determined to be 0.057 μM s^-1^ and 24.7 μM respectively. Catalytic efficiency of probe reduction by NTR (k_cat_/K_m_) was determined to be 2.25 × 10^7^ M^-1^ s^-1^, which is two orders higher than that of luciferin-luciferase reaction (1.07 × 10^5^ M^-1^ s^-1^) [53]. To evaluate if the probe can be used as a reporter of NTR activity, we investigated the effect of the NTR inhibitor dicoumarol (competitive with NADH) on the efficiency of NCL uncaging. A gradual concentration-dependent decrease in signal was observed (Fig 2A) indicating that the uncaging of NCL depends on the activity of NTR. We also assayed quantitative capability of the probe against the amount of NTR by fluorescence and found the detection limit to be 0.15 μg/mL (S2 Fig).

To determine the utility of NCL as a bioluminescent reporter, we measured the light emission from increasing concentrations of NCL (0.25–5 μM) in the presence or absence of NTR and firefly luciferase (Fig 2B). The resulting signal was concentration-dependent and no significant light was produced in the absence of NTR, resulting in high signal to background noise ratios even at relatively low concentrations of the probe in comparison to that previously reported [36,42]. In addition, NCL demonstrated an average 70% conversion into luciferin, the highest uncaging efficiency among existing caged luciferin substrates reported to date [36,42]. We also verified that NTR did not have any effect on the luciferin-luciferase reaction (S4 Fig).

### Imaging of NTR activity in *E*. *coli*

**Fig 3:**
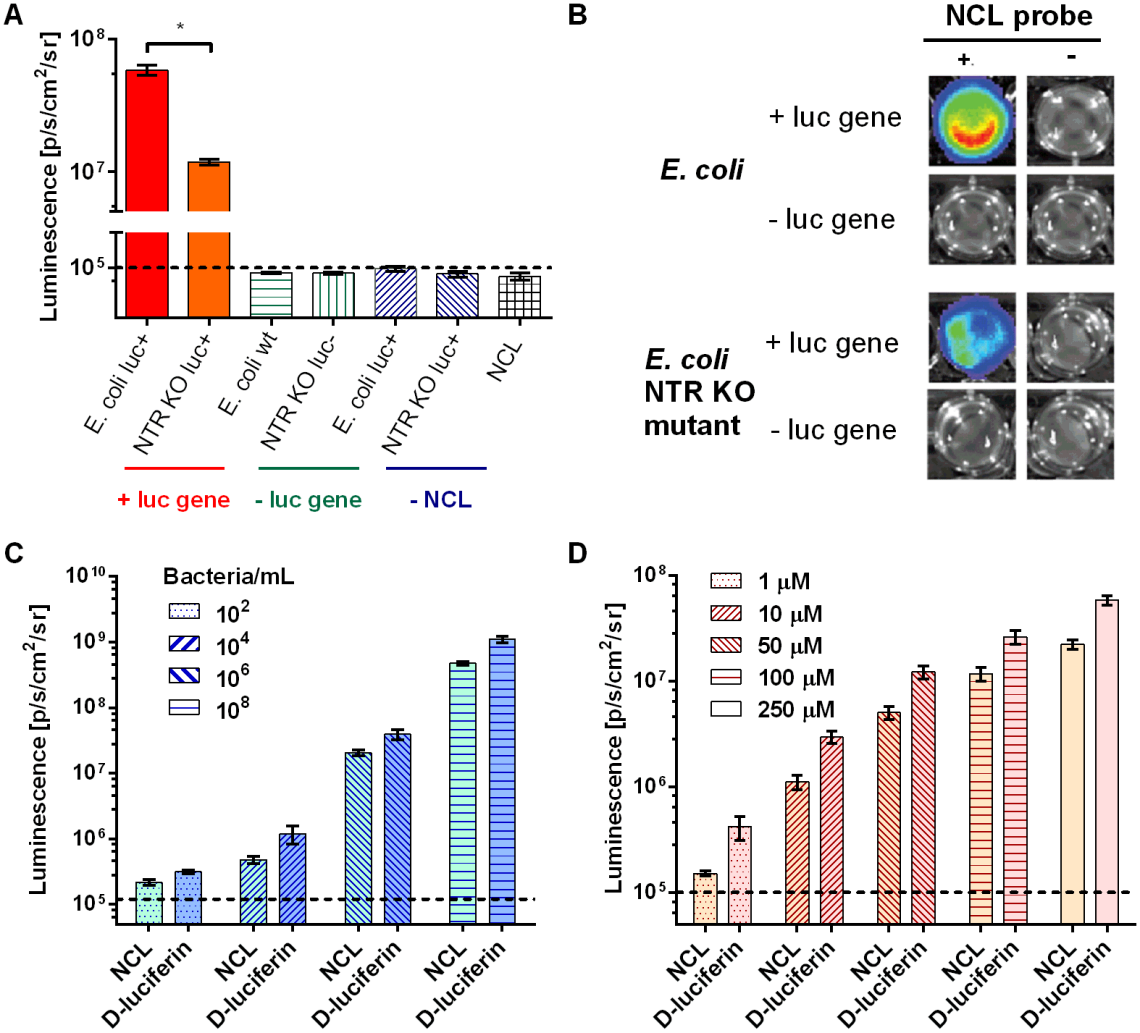
Light production in E. coli by NTR-mediated uncaging of NCL. (A). Light output from NCL (300 μM) after 2 h incubation in luciferase-expressing E. coli (+ luc gene) is significantly higher than in NTR mutant (NTR KO luc+) (*P < 0.001) and in wild type (- luc gene). The dashed line indicates the background. (B). Overlay of a photographic image and bioluminescence from the assay described. (C). Bioluminescence from 100 μM NCL probe and luciferin incubated with various concentrations of luciferase expressing *E*.*coli* AB1157 (10^2^–10^8^ bacteria mL^-1^) for 10 min before imaging. (D). Bioluminescence from luciferase expressing *E*.*coli luc+* (10^8^ bacteria mL^-1^) incubated with various concentrations of NCL or luciferin (1–250 μM) for 10 min before imaging.

We approached the validation of NCL in a live biological system initially utilizing bacteria as a source of both NTR and luciferase. We investigated the potential of NCL probe for imaging NTR in *E*. *coli* naturally expressing NTR [2–4] and engineered to express luciferase. The resulting signal from NCL or luciferin control was compared between several *E*. *coli* strains: 1) *E*. *coli wt*, 2) *E*. *coli* engineered to stably express luciferase (*E*. *coli luc+*), 3) a strain of *E*. *coli* (*NTR KO luc+*) with three well-described NTR genes knocked out (NfsA, NfsB and NemA). First, as shown on Fig 3A, significant signal above background was detected from *E*. *coli luc+* in comparison with *E*. *coli wt*, indicating the need of luciferase presence for light production. Both *wt* and *NTR KO luc+* strains showed similar levels of luciferase expression when treated with luciferin. However with NCL the signal from parent strain was significantly higher than from the NTR mutant strain (*NTR KO luc+*), demonstrating probe selectivity for detection of NTR activity in bacteria. The presence of a signal in wells with *NTR KO luc+* strain indicates that reduction of the nitrofuryl cage is not exclusive or specific to any of the three major *E*. *coli* NTRs (NfsA, NfsB and NemA) and that cage reduction can be achieved at detectable levels in the presence of the remaining NTRs in *E*. *coli*. In E.coli, several nitroreductases (NfsA [2], NfsB [3], YdjA [4]) and reductases (NemA [54]) are characterized, while the exact functions of other reductase proteins remain unclear. Recent studies [4] indicate that *E*. *coli* reductases could also have additional nitroreductase activity.

We also investigated the signal dependence on different numbers of bacteria and significant signals were evident at concentrations of bacteria as low as 10^4^ cells/mL (1.5 × 10^3^ cells/well) (Fig 3C). The average efficiency of probe uncaging in bacteria was calculated to be 35% (Fig 3D).

### Stability profiling

Since an ideal imaging reagent should be non-toxic and stable in biological environments, we next investigated these parameters. The probe did not induce any toxicity in bacteria or mammalian cells (S10 Fig). The half-life of NCL in mouse plasma *in vitro* was determined to be 53.7 h (S6 Fig), which readily permits robust BL imaging *in vivo*. In addition, NCL demonstrated excellent stability to liver microsomes *in vitro* (S7 Fig).

### Imaging of bacterial NTR *in vivo* in a mouse model of intramuscular infection

**Fig 4:**
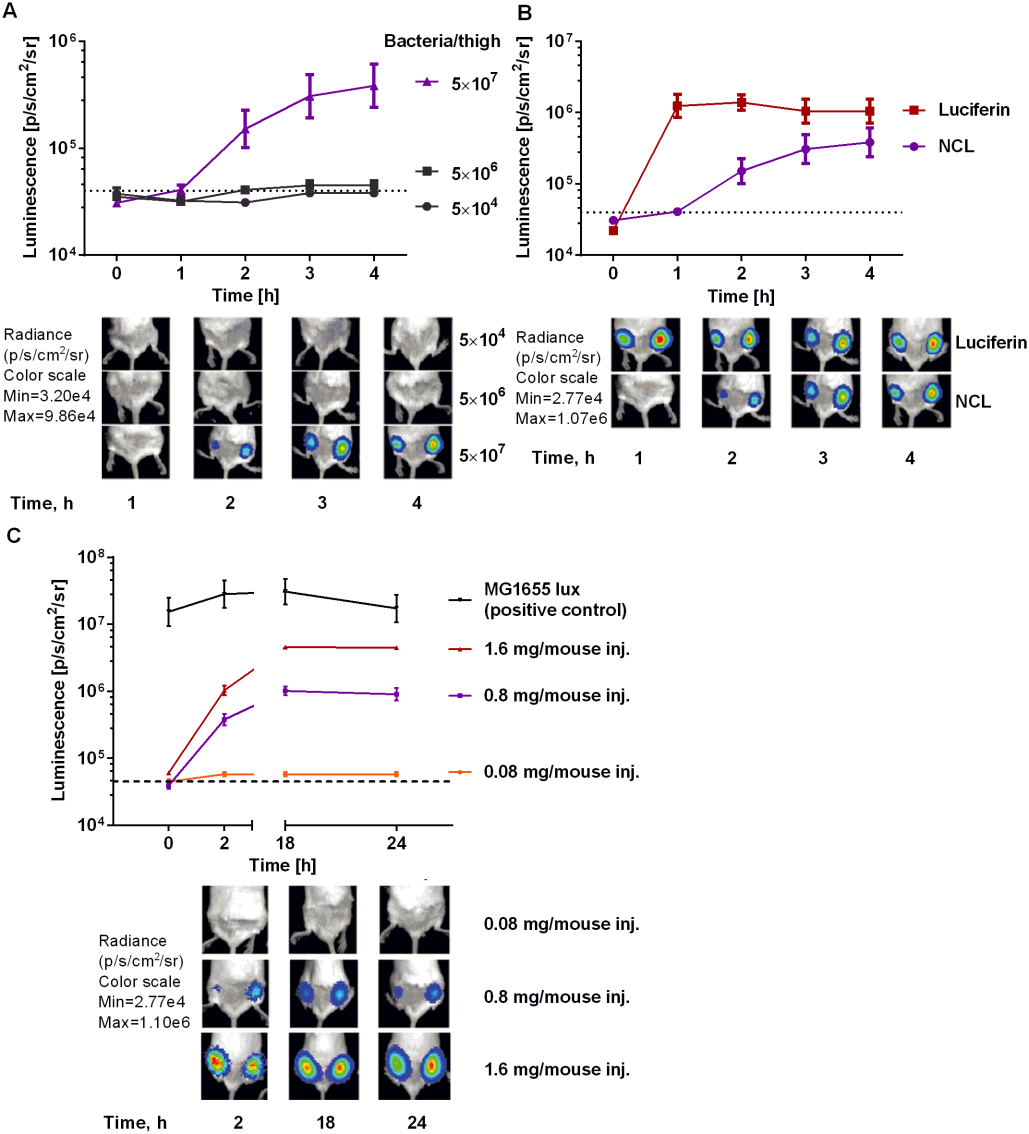
*In vivo* activation of NCL probe by luciferase and nitroredictase expressing *E*.*coli* in a mouse model of thigh infection. (A). Luminescence over 4 h from *E*. *coli luc+* infected quadriceps (5 × 10^4^–5 × 10^7^ bacteria) after IP injection of 0.8 mg NCL probe (200 μL of 10 mM solution in PBS). (B). Luminescence over 4 h from *E*. *coli luc+* infected quadriceps (5 × 10^7^ bacteria) following IP injection of 0.8 mg of probe or 0.63 mg of luciferin (200 μL of 10 mM solution in PBS). (C). Luminescence imaging of mice over 24 h bearing 5 × 10^7^ bacteria, treated with various (0.08, 0.8 and 1.6 mg) NCL probe concentrations (200 μL of 1, 10 and 20 mM solutions of NCL in PBS). As a positive control, mice were injected with equal amounts of *E*. *coli* MG1655 expressing lux luciferase that doesn't require exogenous substrate for light production [44]. The signal was collected over 24 h, n = 3 per group.

The utility, and importantly, the specificity, of the probe *in vivo* was examined in a mouse model of thigh muscle infection, again utilizing bacteria as the source of both NTR and luciferase. Balb/c mice were injected in quadriceps with various numbers of *E*.*coli luc+* (5 × 10^4^–5 × 10^7^), followed by IP injection of NCL 30 min later (Fig 4A). Animals were imaged at various time points over 24 h (Fig 4C). Signal from the probe was detected 20 min post injection lasting for as long as 24 h and correlated with the amount of probe injected. The intensity increased over the first 4 h reaching plateau afterwards. The total photon flux produced during this time was approximately 1/3 of the total flux detected from the mice injected with luciferin, demonstrating high efficiency of uncaging by bacterial NTR *in vivo* (Fig 4B). We also compared the probe kinetics after different administration routes in mouse model of *E*. *coli* intramuscular infection (S8 Fig). Statistical analysis of the data showed no significant difference in signal from IP and IV administration of the probe in this model.

### Evaluation of NCL in NTR expressing cancer cells

**Fig 5:**
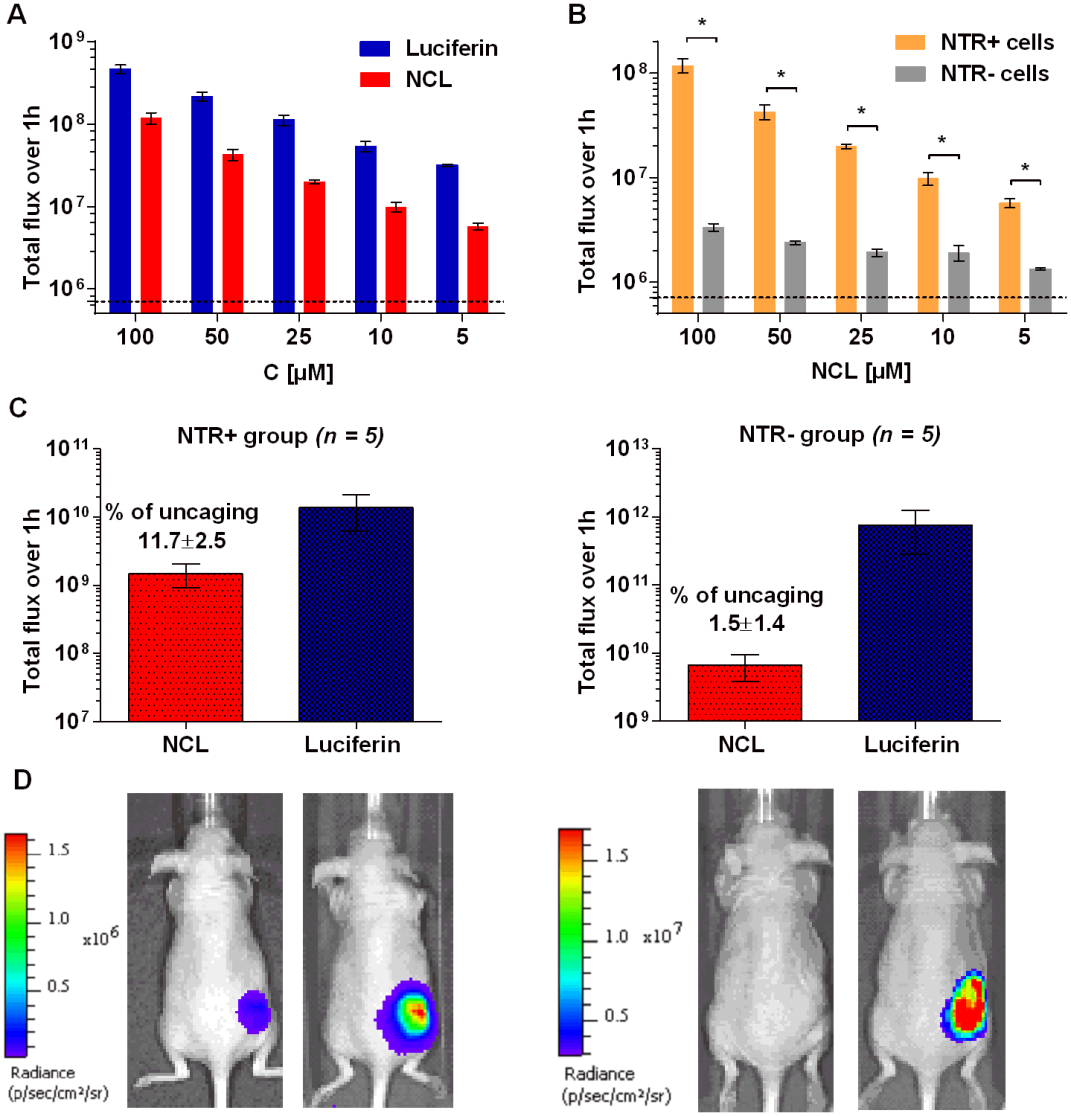
Imaging of NTR activity in cells and in *in vivo* cancer model with NCL. (A) Concentration-dependent uncaging of NCL in MDA-MB-231 NTR+luc+ cancer cells in comparison with luciferin. (B) Selectivity of NTR imaging by NCL in the same cells in comparison with NTR-luc+ cells. The dashed line indicates background (cells only), *P = 0.0001. (C) *In vivo* imaging of NTR activity in subcutaneous NTR+ and NTR- xenografts (n = 5). Total luminescence over 1 h from IP injection of luciferin (1.5 mg) and NCL (1.9 mg). d) Representative images of mice 15 min post injection of luciferin or NCL.

As the next step we applied NCL for imaging of NTR activity in breast cancer cells, stably transfected with NTR and luciferase (MDA-MB-231-NTR+luc+) [25]. The expression of active NTR was confirmed by using the previously described NTR-specific fluorescent CytoCy5S probe (S9 Fig). Addition of different concentrations of NCL to NTR+ cells resulted in rapid concentration-dependent signal increase, with up to 40 times signal-to-noise ratio at the highest concentration used (100 μM) (Fig 5A). Contrary to this, NTR- cells produced much lower signal of equal intensity at all concentrations used (Fig 5B). The difference in brightness between the two cell lines due to different expression levels of luciferase was taken into account by normalizing the signal from NCL to the signal from equimolar quantities of luciferin control. The selectivity of NCL uncaging was also tested in another NTR-/NTR+ cancer cell line and similar results were observed (S11 Fig).

The difference in kinetics of the signal from the probe in bacteria (Fig 4) and mammalian cells (Fig 5) can be explained by the fact that *E*. *coli* naturally expresses several types of nitroreductases, while the mammalian cell line used in our experiments was transfected with one type of nitroreductase.

### Imaging of NTR activity in subcutaneous xenograft model of cancer

In light of these positive results we decided to investigate the utility of NCL as a reporter of NTR activity in a cancer xenograft model in mice, using the same cell lines previously validated *in vitro* (Fig 5). Subcutaneous NTR+ or NTR- xenograft tumors were induced in two groups of mice (n = 5 per group) and were grown to an average volume of approximately 0.25 cm^3^. We chose to implant the NTR+ and NTR- cells in different mice to ensure that the signal produced from the probe is specific to the cell type and doesn't result from luciferin diffusion to the neighboring tumor. Another reason to implant the cells separately was the difference in light emission that could preclude accurate measurements for light sources of different intensity on a single mouse.

When caged luciferin probes are used to measure the activity of a biomolecule in various experimental settings, the light output needs to be calibrated to the amount of luciferase [36,55,56]. In our case this calibration takes into account variations between luciferase levels in tumors from one group to another. Therefore, in order to compare the performance of the probe in two different cancer cell lines with a various level of luciferase expression we normalized the signal from of the probe to the signal from equimolar amount of D-luciferin.

Both groups of mice were first injected IP with luciferin (1.5 mg) to determine the overall light emission from each tumor over 1 h. This signal was later used to calculate percentage of NCL uncaging in order to normalize tumor size and level of luciferase expression from NTR-/+ cell lines. 24 h later, all mice were injected IP with equimolar quantities of NCL (1.9 mg), followed by collection of light over 1 h. Since the residual light from luciferin injection could contribute to the signal from the probe, the absence of residual signal was verified by imaging of mice before probe administration. The level of probe uncaging in NTR+ and NTR- groups was determined by quantifying light production from NCL in each mouse relative to luciferin. As shown in Fig 5C the efficiency of probe uncaging in the NTR+ group was approximately an order of magnitude higher than in the NTR- group (11.7±2.5% and 1.5±1.4% respectively; p = 0.0001), demonstrating the utility of this probe for imaging of NTR activity in tumors.

In a previously reported study of NTR imaging with CytoCy5S fluorescence of orthotopic xenograft tumors, probe activation by bacteria in the gastrointestinal tract (GIT) resulted in background fluorescence in that cancer model requiring fluorescence lifetime gating to differentiate tumor signal from GIT signal [24]. Although we have not presented results of orthotopic tumors, it is anticipated that the bioluminescence based imaging tool in our study, permitting detection of the signal only with coexpression of both luciferase and NTR, would eliminate such issues.

## Conclusions

In conclusion, these results demonstrate that the NCL probe can be effectively used for non-invasive real-time imaging of NTR activity *in vitro*, in live bacteria and mammalian cells, as well as *in vivo*, in preclinical models of cancer and certain bacterial infections (S1 Movie). This novel reagent should significantly simplify screening of prodrugs *in vivo* and accelerate the preclinical development of enzyme-activatable therapeutics for translation into the clinic.

## Supporting Information

S1 Fig Analysis of NCL reduction by *E*. *coli* nitroreductase *in vitro*. (a). UV HPLC profiles (detected at 320 nm) of NCL reduction by NTR over 30 min, peak at 0.4 min corresponds to luciferin (m/z 281), peak at 1.7 min corresponds to NCL (m/z 406). (b). Time course of the conversion of NCL to luciferin, the distribution of reaction products was quantified by HPLC analysis on the basis of absorbance at 320 nm. (c). Absorbance calibration curve for NCL. (d). Pseudo-first-order kinetics of NCL reduction, rate constant 5.8·10^-3^ s^-1^, t_1/2_: 119.5 s.
(TIF)

S2 Fig Kinetic measurements of NCL reduction by NTR *in vitro*. (a). Calibration curve for luciferin fluorescence at λ_ex/em_ = 330/530 nm. (b). Lineweaver–Burk plot of NCL probe reduction (5–50 μM) by NTR from *E*. *coli* NfsA (0.25 μg/mL) in the presence of NADH (500 μM) at 37°C in PBS buffer (pH 7.4). Kinetic parameters K_m_ and V_max_ were determined from Michaelis-Menten model. (c). A plot of fluorescence intensity at λ_330/530_ of different amounts of NCL (1–50 μM) over an incubation time of 15 min with 1 μg/mL of NTR from *E*. *coli* NfsA and 500 μM of NADH at 37°C in PBS buffer (pH 7.4). (d). A plot of fluorescence intensity at λ_330/530_ of 20 μM of NCL, 500 μM of NADH and different amounts of NTR from *E*. *coli* NfsA (0.05–0.2 μg/mL) over an incubation time of 20 min at 37°C in PBS buffer (pH 7.4).
(TIF)

S3 Fig Inhibition of NTR activity by dicoumarol *in vitro*. NCL (20 μM) reduction by NTR (0.5 μg/mL) in the presence of NADH (100 μM) was inhibited by dicoumarol (0 to 200 μM) (Fig 2a). Inhibitory activity of dicoumarol is expressed as a percentage compared to uninhibited control.
(TIF)

S4 Fig Bioluminescent imaging of nitroreductase with NCL in enzyme assay. Light emission from luciferin in the presence and absence of NTR, NTR did not have any effect on the luciferin-luciferase reaction. Total luminescent signal integrated over 30 min from luciferin (0.25–5 μM) with NADH (100 μM), luciferase (60 μg/mL) and NTR (10 μg/mL) compared to the control (without NTR).
(TIF)

S5 Fig Bioluminescence imaging of NTR by NCL in various *E*. *coli* strains. (a). Bioluminescence from 100 μM NCL probe incubated with the wild type AB1157 and luciferase expressing AB1157 CBR *E*. *coli* strains (10^7^ bacteria/mL) for 2 h at 37°C before imaging. The dotted line indicates background, calculated as the average signal from wells containing bacteria only. (b). Bioluminescence from NCL probe and luciferin (10 μM) incubated with luciferase expressing *E*. *coli* AB1157 CBR (parent strain) and AB502NemA CBR NTR KO (mutant strain) (10^8^ bacteria/mL) for 10 min before imaging. A significant difference in signal from the probe is observed between parent and NTR mutant strains (p = 0.0062) while the luciferin signal is the same. (c). Signal kinetics over 1 hour from 100 μM NCL and indicated concentrations of bacteria/mL AB1157 CBR.
(TIF)

S6 Fig NCL *in vitro* stability in mouse plasma. T_1/2_: 53.7 h.
(TIF)

S7 Fig NCL *in vitro* stability in mouse liver microsomes. Testosterone was used as a positive control.
(TIF)

S8 Fig
*In vivo* comparison of NCL kinetics after IP and IV administration in a mouse model of thigh infection (luciferase and nitroredictase expressing *E*. *coli*).Luminescence over 2 h from infected mice that received NCL as a percentage of luminescence from mice that were injected with *E*. *coli* lux (positive control for bacterial number)^5^. Mice were infected with 5x10^7^ *E*. *coli*, NCL was administered by IV or IP injection, n = 2–4 per group. Statistical analysis was performed using a two way ANOVA with Bonferroni post-test showing no significant difference between IV and IP administration of NCL. The error bars were calculated using the following equation: $\frac{\text{Luminescence SEM}}{\text{Average lux}}\times 100.$
(TIF)

S9 Fig Fluorescent imaging of NTR with CytoCy5S probe in MDA-MB-231-NTR-Fluc-EGFP and MDA-MB-231-luc cells. (a). The cells were incubated with 400 ng/mL of CytoCy5S in HBSS or HBSS only (control) for 1.5 h, washed with HBSS once and imaged for fluorescence (excitation 640 nm, emission 700 nm) in IVIS Spectrum. Error bars are ±SD of three wells, ***P = 0.0006. (b). Image overlay of a photographic image and epi-fluorescence from the well plate used in the assay.
(TIF)

S10 Fig The CytoTox-Glo Cytotoxicity Assay (Promega). MDA-MB-231-luc cells were treated with various concentrations of NCL for 1 h and the level of ATP was measured using CytoTox-Glo reagent.
(TIF)

S11 Fig Imaging of NTR with NCL in stably transfected cancer cells MDA-MB-231^*GFP+Luc+NTR+*^ compared to the control cells (MDA-MB-231^*GFP+Luc+*^). (a). Bioluminescence from NCL (50–5 μM) 15 min after addition to the cells. (b). Luciferin signal (50–5 μM) 15 min after addition to the cells confirmed equal levels of luciferase in both cell lines. Imaging was performed for 1 h, a single image presented here is at 15 min time point.
(TIF)

S12 Fig Imaging of NTR in subcutaneous xenograft model of cancer. Signal kinetics over 1 hour from luciferin and NCL in NTR+ and NTR- groups (n = 5).
(TIF)

S1 File Contains chemical materials and methods, synthesis, spectra and general methods. (DOCX)

S1 Movie Animation movie demonstrates the concept of the study, probe design and applications. (AVI)
