# Supplementary material for: Development of a Bioluminescent Nitroreductase Probe for Preclinical Imaging
Source: PLoS One. 2015 Jun 25;10(6):e0131037. doi: 10.1371/journal.pone.0131037 (PMC4482324; doi:10.1371/journal.pone.0131037)

**Supporting Information for:**

**Development of a Bioluminescent Nitroreductase Probe for Preclinical Imaging**

Anzhelika G. Vorobyeva^1^, Michael Stanton^2^, Aurélien Godinat^1^, Kjetil B. Lund^3,4^, Grigory G. Karateev^1^, Kevin P. Francis^5^, Elizabeth Allen^6^, Juri G. Gelovani^7^, Emmet McCormack^3,4^, Mark Tangney^2^, Elena A. Dubikovskaya^1*^

^1^ School of Basic Sciences, Institute of Chemical Sciences and Engineering, Swiss Federal Institute of Technology of Lausanne, Lausanne, Switzerland

^2^ Cork Cancer Research Centre, University College Cork, Cork, Ireland

^3^ Department of Clinical Science, University of Bergen, Bergen, Norway

^4^ Department of Internal Medicine, Hematology Section, Haukeland University Hospital, Bergen, Norway

^5^ PerkinElmer, Alameda, California, United States of America

^6^ School of Life Sciences, Swiss Institute for Experimental Cancer Research (ISREC), Swiss Federal Institute of Technology of Lausanne, Lausanne, Switzerland

^7^ Department of Biomedical Engineering, College of Engineering and School of Medicine, Wayne State University, Detroit, Michigan, United States of America

* Corresponding author

E-mail: [elena.dubikovskaya@epfl.ch](mailto:elena.dubikovskaya@epfl.ch) (EAD)

**Content:**

Chemical Materials and Methods S3

Chemical Synthesis S3

General Methods S5

References S6

Spectra S7

**Chemical Materials and Methods**

Chemicals were purchased from ABCR GmbH, Acros organics, AppliChem GmbH, Sigma-Aldrich, and were used as received. Firefly luciferase, *E. coli* nitroreductase NfsA, mouse male liver microsomes were purchased from Sigma-Aldrich. Mouse plasma was purchased from Harlan Laboratories. Analytical thin layer chromatography was performed using aluminum-backed SiO_2_ TLC plates from Merck. HPLC analysis was performed on Agilent Infinity 1260 HPLC system (Agilent, Santa Clara, CA) with SunFire C18 column (2.6x20 mm, 3.5 µm, Waters) using degassed HPLC gradient grade solvent from Fisher Chemicals (Loughborough, UK) and Millipore water. The products of the reaction were initially analyzed by Agilent 6120 Quadrupole LC/MS system (Agilent, Santa Clara, CA), directly connected to HPLC. Nuclear magnetic resonance (^1^H, ^13^C NMR) data were acquired on a Bruker AV-400 spectrometer. NMR chemical shifts are reported in the standard δ notation of parts per million using the peaks of residual proton and carbon signals of the solvent as internal references. Splitting patterns are designated as s (singlet), d (doublet), dd (doublet of doublets), t (triplet), m (multiplet). Coupling constants (*J*) are reported in hertz. HRESI-MS measurements were conducted at the EPFL ISIC Mass Spectrometry Service using Micro Mass QTOF Ultima (Waters Corp., Milford, MA).

**Chemical Synthesis**

**(5-Nitrofuran-2-yl)methanol (2)**

Compound **2** was synthesized using a method described in the literature^1^. 5-Nitro-2-furaldehyde (2.0 g, 14.2 mmol) was dissolved in THF (25 mL). Sodium borohydride (0.45 g, 14.2 mmol) in water (2 mL) was added slowly dropwise to the THF solution at 0°C. After the addition, the ice bath was removed and the reaction mixture was left to stir at room temperature for 1 h 30 min. THF was evaporated, 20 mL of water was added to the reaction mixture and extracted with ethyl acetate (3 x 30 mL). The combined organic extracts were washed with saturated NaCl solution, dried (Na_2_SO_4_) and evaporated. After evaporation the desired product was obtained as a dark-yellow liquid (1.19 g, 59%).

**6-((5-Nitrofuran-2-yl)methoxy)benzo[d]thiazole-2-carbonitrile (4)**

To a solution of compound **2** (0.25 g, 1.75 mmol), 2-cyano-6-hydroxybenzothiazole, **3** (0.277 g, 1.57 mmol), and triphenylphosphine (0.642 g, 2.45 mmol) in anhydrous THF (7 mL) under N_2_, was added a solution of 1,1'-(azodicarbonyl)dipiperidine (ADDP) (0.617 g, 2.45 mmol) in anhydrous THF (4 mL) dropwise at 0°C. After 1.5 h, the reaction mixture was placed at room temperature and stirred for 18 h. The solvent was evaporated and crude mixture was purified using Biotage (SNAP C18 12g column, H_2_O:THF, 3-100% THF over 30 min). Purification yielded 0.226 g (43%) of compound **4**. ^1^H NMR (400 MHz, (CD_3_)_2_CO) δ = 8.03 (d, J=9.1 Hz, 1H), 7.85 (d, J=2.6 Hz, 1H), 7.40 (d, J=3.7 Hz, 1H), 7.31 (dd, J=9.1, 2.6 Hz, 1H), 6.90 (d, J=3.7 Hz, 1H), 5.29 (s, 2H). ^13^C NMR (101 MHz, (CD_3_)_2_CO) δ = 159.60, 154.15, 153.39, 148.38, 138.55, 135.36, 126.72, 119.84, 114.72, 114.10, 113.45, 106.37, 63.41. HRMS: calculated for [C_13_H_7_N_3_O_4_S]^+^  302.0229, found 302.0236.

**2-(6-((5-nitrofuran-2-yl)methoxy)benzo[d]thiazol-2-yl)-4,5-dihydrothiazole-4-carboxylic acid (5)**

To a solution of compound **4** (0.048 g, 0.16 mmol) in THF (1.5 mL) was added a solution of D-cysteine (0.029 g, 0.24 mmol) in degassed PBS (1.2 mL) under N_2_. After 1 h, solvent was evaporated and crude material was purified by preparative HPLC (C8 column, H_2_O+5mM HCOONH_4_:ACN, 5-95% ACN over 30 min), affording pure product **5** (0.044 g, 68%). ^1^H NMR (400 MHz, CD_3_CN) δ = 7.97 (d, *J*=9.0 Hz, 1H), 7.63 (d, *J*=2.6 Hz, 1H), 7.41 (d, *J*=3.7 Hz, 1H), 7.22 (dd, *J*=9.1, 2.6 Hz, 1H), 6.83 (d, *J*=3.7 Hz, 1H), 5.27 (t, *J*=9.2 Hz, 1H), 5.20 (s, 2H), 3.71 (d, *J*=6.2 Hz, 1H), 3.68 (d, *J*=5.5 Hz, 1H). ^13^C NMR (101 MHz, (CD_3_)_2_CO) δ = 171.41, 166.20, 159.83, 154.52, 158.51, 153.32, 149.29, 138.55, 126.05, 118.26, 114.51, 113.45, 106.71, 79.34, 63.33, 35.59. HRMS: calculated for [C_16_H_11_N_3_O_6_S_2_]^+^ 406.0168, found 406.0171.

**General Methods**

**Nitroreductase assay.** The reduction of NCL probe by *E. coli* nitroreductase (NTR) was monitored by HPLC analysis. The reaction solution containing 700 µL of PBS (pH 7.4), 100 µL of 10 mM NADH, 100 µL of 10 µg/mL NTR and 100 µL of 10 mM NCL solution in PBS (pH 7.4) was performed at 37 °C. Aliquots of 100 µL were taken from reaction mixture at 0, 0.5, 1, 3, 5, 7, 9 and 30 min, and were quenched with 100 µL of acetonitrile. The samples were analyzed by HPLC. Kinetics of the reaction was calculated assuming pseudo-first-order kinetics and a plot of ln of probe concentration versus time was established. The half-life of reduction was calculated on the basis of the disappearance of the substrate assuming pseudo-first-order kinetics.

**HPLC analysis.** HPLC analysis was performed on Agilent Infinity 1260 HPLC system (Agilent, Santa Clara, CA) with SunFire C18 column (2.6 × 20 mm, 3.5 µm, Waters) using degassed HPLC gradient grade solvent from Fisher Chemicals (Loughborough, UK) and Millipore water. The products of the reaction were initially analyzed by Agilent 6120 Quadrupole LC/MS system (Agilent, Santa Clara, CA), directly connected to HPLC. NCL and luciferin were detected at 320 nm, testosterone was detected at 240 nm. Conditions of the method were the following: mobile phase 5 mM HCOONH_4_:ACN, 5:95% ACN over 2 min, the injection volume 3 µL and the flow rate 1 mL/min. Standard curve for NCL was constructed by plotting the peak areas vs. diluted concentrations (in a range of 1- 50 µM) of stock solutions of the compound.

***In vitro* stability in mouse plasma.** The *in vitro* stability of NCL was studied in mouse plasma (Harlan Laboratories) according to the procedure described in the literature^2^. The reactions were initiated by the addition of 100 µL of 1mM solution of NCL in PBS (pH 7.4) to 400 µL of preheated plasma solution to yield a final concentration of 200 µM. The assays were performed in a shaking water bath at 37 °C. Samples (50 µL) were taken at 0, 15, 30, 45, 60 min, 2, 6 and 24 h and added to 150 µL of ice-cold methanol. The samples were subjected to brief vortex mixing and centrifugation for 10 min at 14000 rpm. The clear supernatants were analyzed by HPLC. The *in vitro* plasma half-life (t_1/2_) was calculated using the expression t_1/2_=ln2/b, where b is the slope found in the linear fit of the natural logarithm of the fraction remaining of the NCL vs. incubation time.

***In vitro* mouse liver microsome stability assay.** The assay was performed according to the procedure described in the literature^3^. The reaction mixture contained 390 µL of PBS (pH 7.4), 10 µL of microsomes (20 mg/mL) with or without 50 µL of 10 mM NADPH. The reaction was initiated by the addition of 50 µL of 1 mM solution of NCL in PBS (pH 7.4) to the reaction mixture. At 20, 40 and 60 min 100 µL aliquots were taken and quenched with 150 µL of ice-cold methanol. The samples were subjected to brief vortex mixing and centrifugation for 10 min at 14000 rpm. The clear supernatants were analyzed by HPLC. The microsomal activity was monitored using testosterone as a positive control.

**Bacterial viability assay.** *E. coli* K-12 AB1157 CBR and *E. coli* K-12 AB502NemA CBR were cultured aerobically overnight. Bacteria were then subcultured and divided into two groups (untreated and probe treated) in triplicate in fresh media and grown until cultures reached OD_600_ of 0.2. At this point, NCL probe treated group was treated with 100 µM probe. At hourly time points, aliquots from all cultures were subjected to serial dilution before plating on Amp selective agar to determine cfu/mL values. Cultures were also tested for changes in luminescence over time to identify if any differences existed between bacteria grown alone and bacteria grown in the presence of the probe.

**Cell lines and cell culture.** Cell lines used for *in vitro* experiments (Figure S11) were generated as described in the work of McCormack et al.^4^ The triple negative mammary carcinoma MDA-MB-231*^wt^* and MDA-MB-231*^GFP+Luc+^* were kindly provided by Prof. James Lorens (University of Bergen, Norway). The MDA-MB-231*^GFP+Luc+^* cells were transfected with the retroviral expression vector L149 pTra Puro2AGFP2ALuciferase2NTR (Entrez: EU753858) containing genes expressing *E*. *coli* enzyme Nitroreductase (NTR) and green fluorescence protein (GFP), and was named MDA-MB-231*^GFP+Luc+NTR+^*.^4^ The MDA-MB-231 cell lines were maintained in Dulbecco’s Modified Eagle’s Medium. The media were supplemented with 10% heat-inactivated FBS (HyClone, Thermo Scientific), 1% penicillin/streptomycin (PS; Sigma-Aldrich) and 1% L-glutamin (Sigma-Aldrich). The cells were incubated in a humidified atmosphere at 37°C in 5% CO_2_. Authentication of all cell lines was done by DNA fingerprinting using the AmpFISTR Profiler Plus PCR Amplification kit (Applied Biosystems) in April 2013.^4^

**Bioluminescent imaging of nitroreductase by NCL in stable cell lines.** The assay shown in Figure S11 was performed as described below. The cells were seeded out in a 96 well plate in triplets with descending concentrations of compounds. The amount of media in each well was set to 100 µL. One 96 well plate contained triplets of NCL probe, luciferin, cells only and media only. These 96 well plates with cells were set to rest for two hours after seeding before imaging. Descending concentrations of NCL probe or luciferin (10 µL) were added to each well with a mechanical 1- 10 µL multichannel Finnpipette F2 (Thermo Labsystems, Milford, MA, USA). Bioluminescence images were acquired using KODAK In Vivo Multispectral Imaging System FX (Carestream Healt, NY, US) every two minutes for 1 h without filters. The images were acquired over 90 s with 30 s of rest between each acquisition. Images were analyzed using Carestream Molecular Imaging Software (version 5.0.6.20).

**Cell viability assay.** MDA-MB-231 (NTR–luc+) cells were plated at a density 1 × 10^4^ cells/well in a 96 well plate, the next day cells were treated with different concentrations of NCL probe (5–150 µM solutions in cell culture medium) for 1 h, control group was incubated with medium only. CytoTox-Glo™ reagent was prepared as indicated and 80 µL of solution was added to each well. After 20 min luminescence was measured using a plate reader Tecan Infinite M1000 (Tecan Austria GmbH) with integration time 0.5 s.

**References**

1. Borch RF, Liu J, Schmidt JP, Marakovits JT, Joswig C, et al. (2000) Synthesis and Evaluation of Nitroheterocyclic Phosphoramidates as Hypoxia-Selective Alkylating Agents. *J Med Chem* 43: 2258–2265.

2. Konsoula R, Jung M (2008) In vitro plasma stability, permeability and solubility of mercaptoacetamide histone deacetylase inhibitors. *Int J Pharm* 361: 19–25.

3. Hill JR (2004) In Vitro Drug Metabolism Using Liver Microsomes. In: Enna SJ, Williams M, Ferkany JW, Kenakin T, Porsolt RD, et al., editors. *Current Protocols in Pharmacology*. Hoboken, NJ, USA: John Wiley & Sons, Inc.

4. McCormack E, Silden E, West R, Pavlin T, Micklem D, Lorens J, Haug B, Cooper M and Gjertsen B (2013) Nitroreductase, a new-infrared reporter platform for in vivo time-domain optical imaging of metastatic cancer. *Cancer Res*, 1276-1286.

5. Cronin M., Akin A. R., Collins S. A., Meganck J., Kim J.-B., Baban C. K., Joyce S. A., van Dam G. M., Zhang N., van Sinderen D., O'Sullivan G. C., Kasahara N., Gahan C. G., Francis K. P., Tangney M. (2012) *PLoS ONE*, 7(1), e30940.


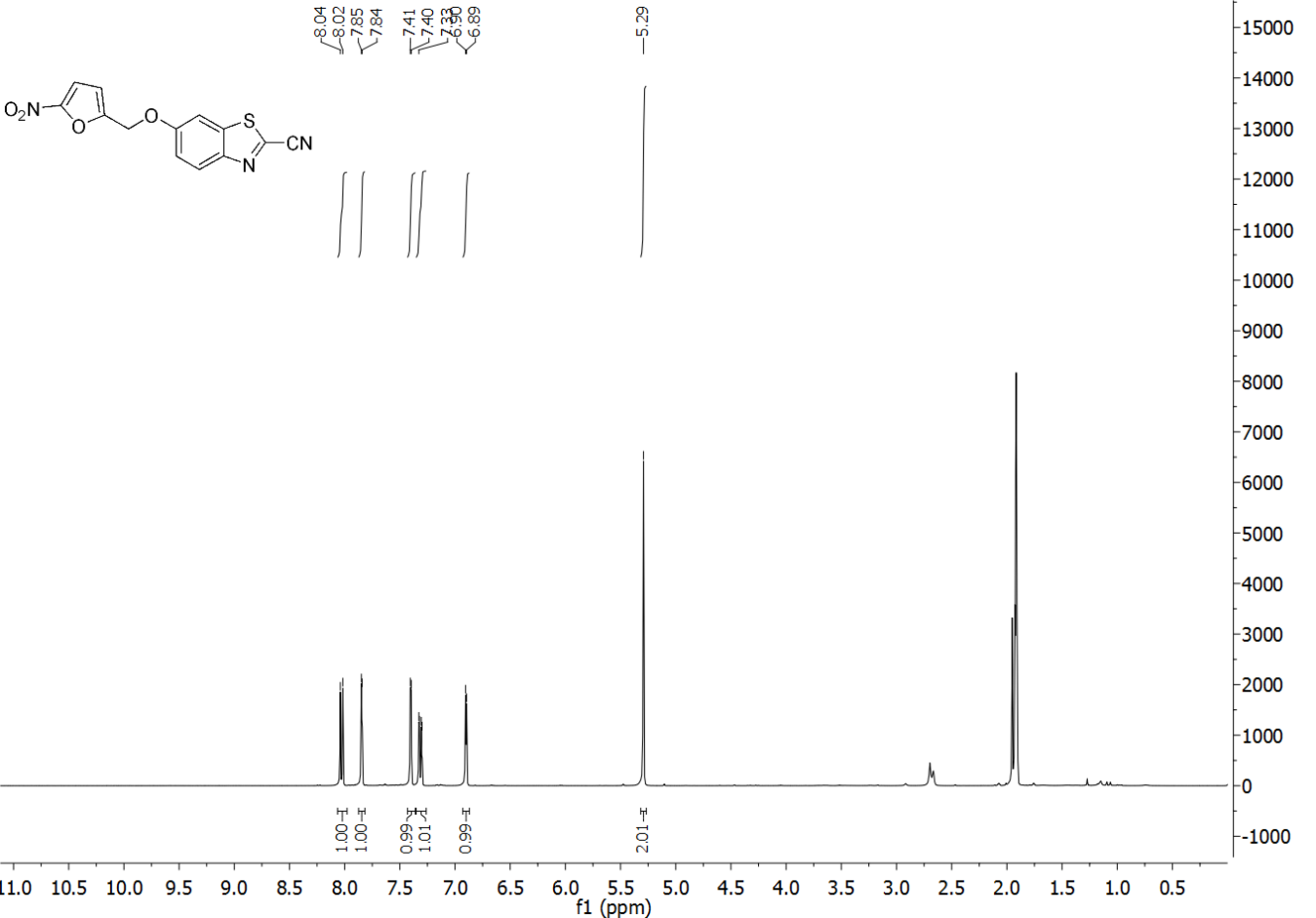
**Spectrum ^1^H of compound 4.**


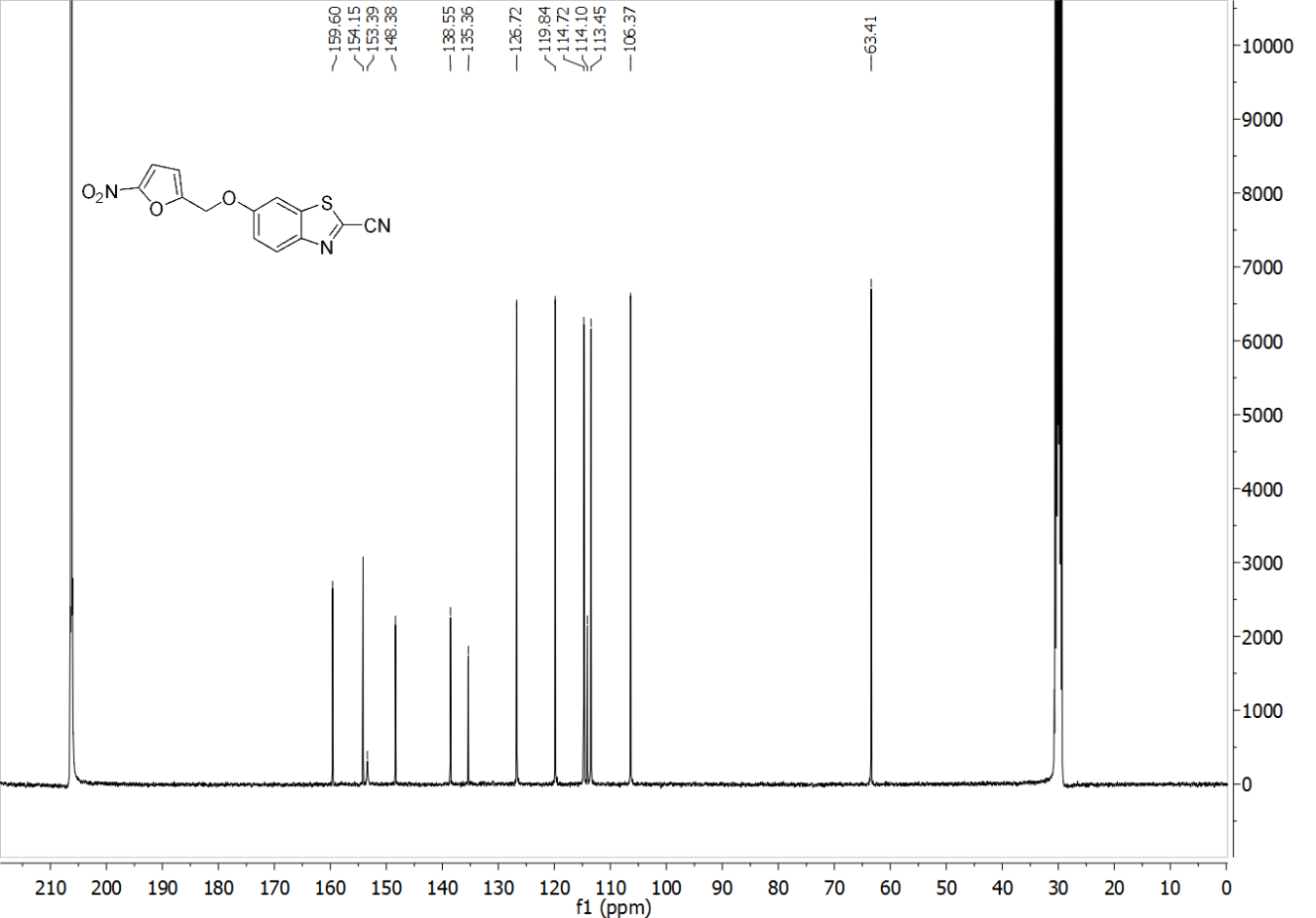
**Spectrum ^13^C of compound 4.**

**Spectrum ^13^C-DEPT-135° of compound 4.**


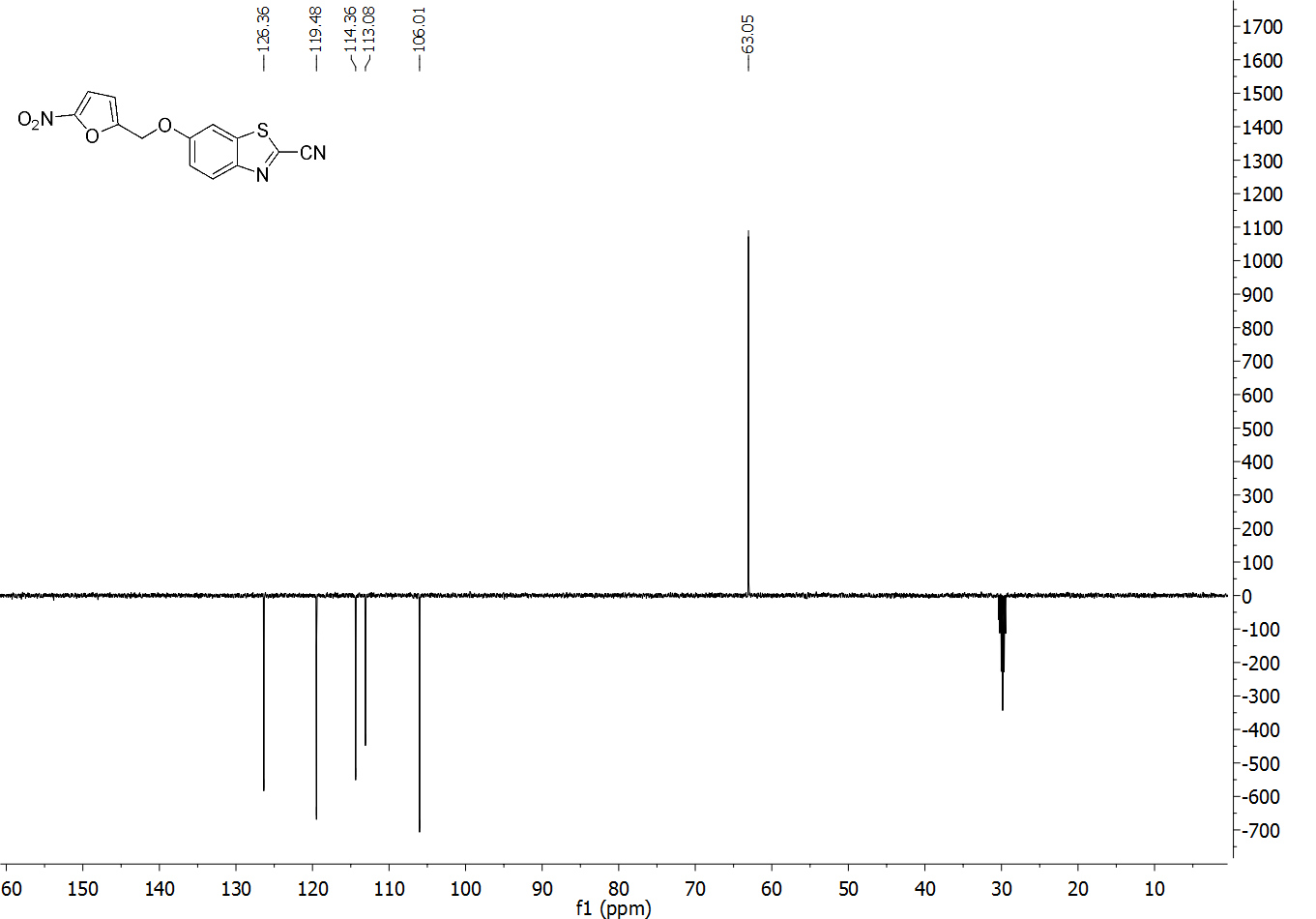


**Spectrum ^1^H of compound 5.**

**
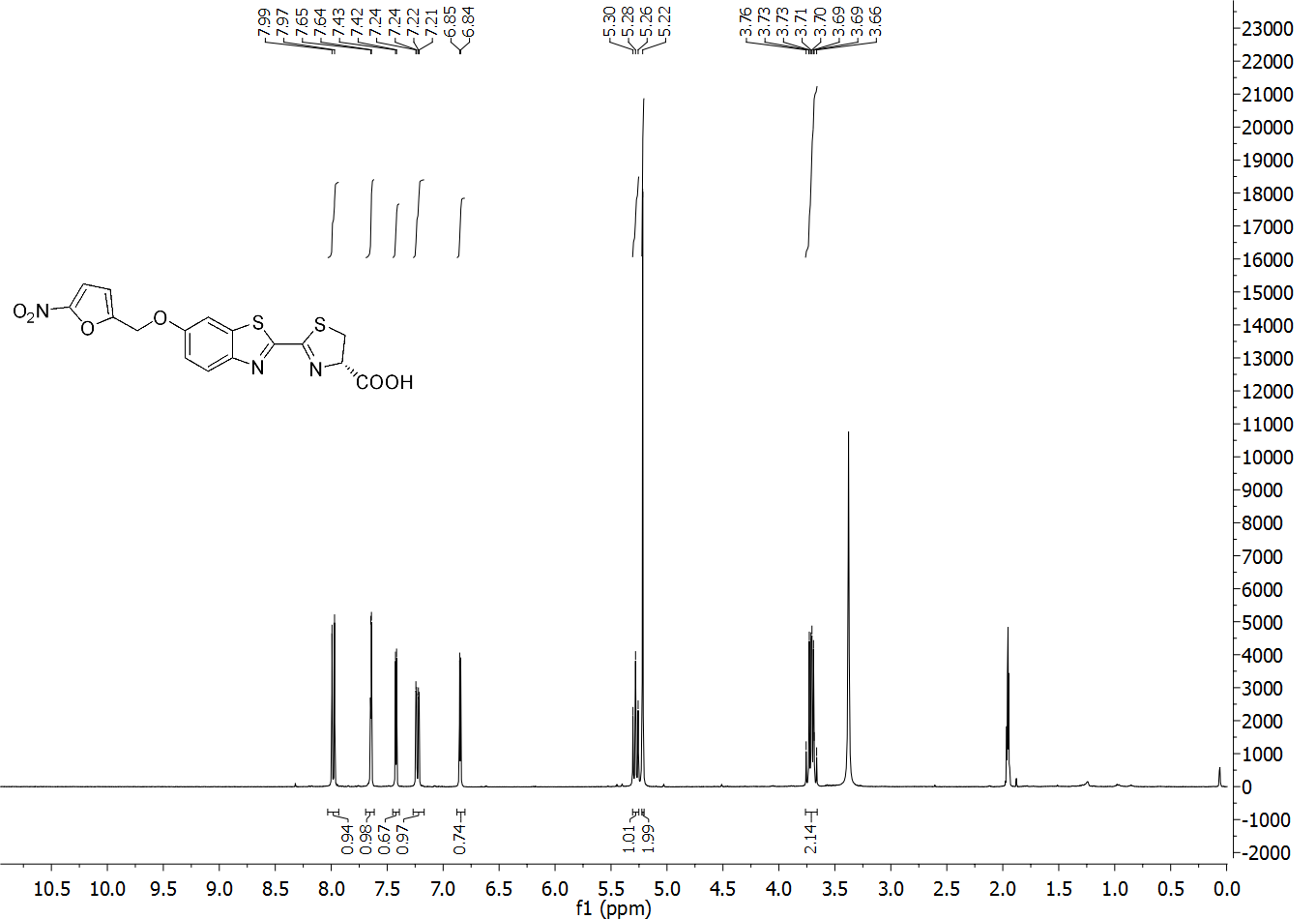
**

**Spectrum ^13^C of compound 5.**


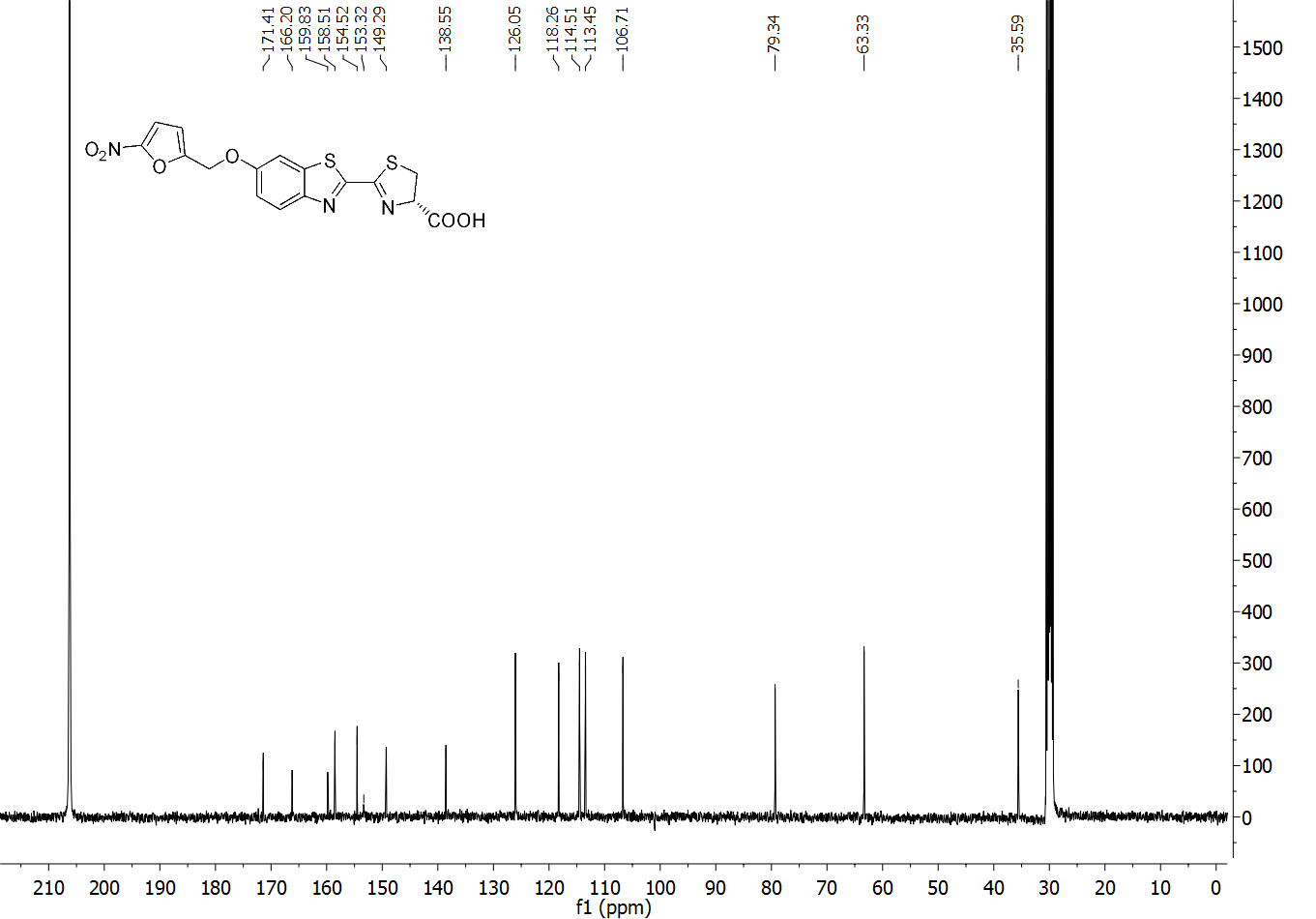

Supplement: S1 File — (DOCX) [file pone.0131037.s013.docx]
